# Supplementary material for: Are carbon emissions trading and green financial instruments synergistic? -Comprehensive quantitative research based on content analysis
Source: PLoS One. 2024 Mar 7;19(3):e0298601. doi: 10.1371/journal.pone.0298601 (PMC10919601; doi:10.1371/journal.pone.0298601)
Supplement: S1 Data — (ZIP) [file pone.0298601.s001.zip › Data/地级市Z_F工作报告.zip!/║╙─╧/─╧╤⌠╩╨/└┤╫╘╣1⁄2╓┌║┼í╛┬φ┐╦╩2╛▌═°í┐_2017.docx]

各位代表：

现在，我代表市人民政府，向大会报告工作，请予审议，并请各位政协委员和其他列席人员提出意见。

一、2016年工作回顾二号首长

过去的一年是不平凡的一年。一年来，面对复杂的外部环境和繁重的发展任务，在省委、省政府和市委的坚强领导下，我们紧密地团结在以习近平同志为核心的党中央周围，带领全市人民，深入贯彻落实中央和省委、省政府各项决策部署，以新发展理念为引领，主动适应经济发展新常态，坚持“四一二”工作大局和“一优一高一稳定”工作方针，奋力躬耕，攻坚克难，圆满完成了市五届人大三次会议确定的主要目标任务，全市经济社会平稳健康发展，实现了“十三五”良好开局。

(一)稳增长、保态势，经济运行稳中有进

把稳增长、保态势、调结构、防风险作为重要任务，统筹谋划，精心部署，长短结合，综合施策，经济运行总体平稳、稳中有进。

1.经济平稳增长。狠抓投资拉动，强化产业支撑，主要经济指标增速高于全省平均水平。全市生产总值增长8.5%、高于全省0.4个百分点，固定资产投资增长16.7%，规模以上工业增加值增长8.8%，一般公共预算收入增长8.3%，社会消费品零售总额增长11.9%，居民人均可支配收入增长8%，居民消费价格上涨1.4%。

2.结构持续优化。着力推进供给侧结构性改革，“三去一降一补”取得积极成效，转型升级步伐加快。农业结构稳中调优，粮食生产保持稳定，畜牧业加快发展，绿色、有机农产品基地面积和认证数量均居全国地级市之首。工业结构持续优化，装备制造、纺织服装、绿色食品三大产业稳步发展，县区超百亿产业集群达到5个，高技术产业和高成长性产业分别增长16%和9.8%，规模以上工业实现利润增长4.2%。服务业加快发展，增长10.3%，成为拉动经济增长的重要因素。旅游业持续快速发展，全年接待游客4300万人次，实现旅游综合收入228亿元，新创建4A级景区2家。新业态新模式迅猛发展，全市注册电商达到2576家，电子商务交易额增长68.8%。三次产业结构调整为15.1∶44.8∶40.1。

3.“三区”稳步提升。全市产业集聚区完成固定资产投资1600亿元，实现规模以上工业主营业务收入2400亿元，新能源产业集聚区入选全省“十强”，桐柏县安棚化工专业园区晋升为产业集聚区。西峡县商务中心区、内乡县特色商业区分别入选全省“十快”和“十先进”。

4.风险有效防控。坚持依法依规、打防结合，积极防范处置非法集资风险，千方百计减少群众损失。有效化解房地产、企业互保等领域潜在风险。

(二)抓“双创”、保生态，城乡环境明显改观

以创建国家卫生城市、全国文明城市为抓手，以环境治理为重点，城乡环境和市民素质同步提升，大美南阳建设迈出新步伐。

1.“双创”攻坚卓有成效。把“双创”作为提升城市形象、居民素质和人民幸福指数的重要载体，全面发动、全民参与、全力争创。硬化美化4000多条主次干道和背街小巷，建成19个精品街区，改造提升23个游园公园；新增186台新型环卫车，新建改造353座公厕和垃圾中转站；新建提升各类市场125个，规范提升1.1万家食品餐饮单位，“五小”达标率超过98%；初步建立了健康教育网络，建成了一批综合性文化活动中心；在全市范围内广泛开展文明创建和志愿者服务活动；火海救人英雄王锋当选“感动中国”十大人物；顺利通过国家“创卫”暗访评估和“创文”年度测评，城乡面貌、文明程度明显提升，人民群众广泛受益。

2.城乡建设提速提质。内河综合治理全面推进，温凉河综合治理工程基本完工，梅溪河、三里河、护城河、汉城河综合治理工程加快推进，溧河、十二里河综合治理工程开工建设。白河城区段综合整治工程取得阶段性成果。中心城区17条市政道路建成通车。海绵城市试点规划编制完成。“三馆一院”、南水北调精神教育基地主体工程基本完工。新增城市公交200辆。数字城管等平台建成投用。县域城镇体系提质扩容，成为吸纳农业转移人口的主阵地。乡村清洁工程和美丽乡村建设深入推进，农村人居环境质量不断提升。

3.生态环境明显改善。把环境治理作为紧迫任务，狠抓重点领域污染防治，环境保护和生态建设力度加大。全力打好大气污染防治攻坚战，一手抓治理、一手抓管控，实施“六控一应对”，开展8项专项整治，中心城区空气优良天数达到202天；PM10、PM2.5浓度同比分别下降13.1%和14.9%，各项指标均优于上年、优于全省、位居前列。深入实施“五水共治”，14个责任目标断面水质平均达标率95.2%，全市集中饮用水水源全部达标。南水北调中线调水两周年累计输水60亿立方米。加强生态建设，成功申报总投资366.7亿元的国家储备林基地项目，积极开展国家森林城市创建工作，全面停止天然林商业性采伐，完成营造林182万亩。全市人民向往的蓝天白云、青山绿水的美好愿景正在加快实现。

(三)增动能、强支撑，转型发展基础更加坚实

以改革开放创新激发活力动力，以基础能力建设强化支撑，发展后劲和势能不断蓄积。

1.重点改革不断深化。全面完成“放管服”、国企改革年度任务，取消、调整、下放各类审批事项81项，265项前置审批事项改为后置审批。财税、金融、投融资等领域改革取得新进展，跻身第二批创建国家信用体系建设示范市。农村改革、医药卫生体制改革深入推进，农村土地确权登记颁证工作基本完成，方城、西峡率先建立医院法人治理结构。机关事业单位养老保险、综合执法体制、出租车行业等改革有序实施。

2.开放招商稳步推进。卧龙综合保税区封关运行。京宛对口协作全面展开，40个重大合作项目进展顺利。全市新引进亿元以上项目368个，实际利用外资6.37亿美元，实际到位省外资金677亿元。成功申办2019年世界月季洲际大会，为我市扩大开放、提升品位、展示形象提供了又一重要机遇和平台。

3.科技创新初见成效。争取省以上科技项目74个，38个“百项科技创新项目”成功结项。新增省级以上创新平台12个，中关村科技产业园、京宛科技合作等平台效应初步显现，与郑州大学等高校合作进展顺利。南阳创业大街等一批众创空间建成运行，南阳三融众创空间等创新创业载体获得国家、省认定命名，成功举办全市首届创新创业大赛。

4.基础能力显著提升。宁西铁路二线建成通车，郑万高铁、蒙华铁路建设进度居全线前列，高铁片区规划同步启动，南阳火车站改扩建工程进展顺利，宁西高铁进入中原城市群发展规划，呼南高铁豫西通道列入部省工作计划；新开通厦门、兰州航班，南阳机场旅客吞吐量突破60万人次；周南高速、栾西高速、中心城区东环路开工建设；干线公路、农村公路完成投资32亿元；加强信息基础设施建设，入选“宽带中国”示范城市。内乡煤电运一体化、南召天池抽水蓄能电站等重大能源项目加快建设，镇平玉都500千伏变电站建成投用，风电、光伏发电累计并网发电规模达到39.5万千瓦。唐河复航工程、罗汉山大型水库列入国家“十三五”专项规划。

(四)惠民生、促和谐，群众获得感持续增强

努力增进人民福祉，办好民生实事。全市财政民生支出438亿元，占一般公共预算支出的79.8%，共享发展取得新成效。

1.脱贫攻坚扎实有效。坚持精准识别、精准施策、精准退出，抓好转移就业、产业帮扶、易地搬迁、社会保障、社会救助五项重点工作，强化教育、医疗、交通、水利、电力五大基础建设，各级财政安排专项扶贫资金9.9亿元、同比增长1.55倍，245个贫困村脱贫摘帽，12.7万农村贫困人口摆脱了贫困。

2.就业创业态势良好。实施就业促进和创业引领计划，开展职业技能培训24.1万人次，高校毕业生就业保持稳定，失业人员再就业、就业困难人员就业完成年度目标，城镇新增就业8.5万人，农村劳动力新增转移就业10.3万人， 2.7万名外出务工人员返乡创业。

3.社会保障持续加强。各项社会保险参保达到853.8万人次。新农合参合率达到99.6%，人均筹资标准提高到540元。启动整合城乡居民医保。社会保险费改由地税机关征收。城乡低保月人均补助水平分别提高到240元和132元。农村特困人员集中和分散供养标准分别达到每人每年4000元和3000元。新解决20万名农村居民饮水安全问题。新开工保障性住房9347套，改造农村危房13384户。

4.社会事业全面进步。提高教育供给能力，薄弱学校办学条件得到改善。全市新建、改扩建幼儿园424所、中小学840所，新增学位29万个，其中中心城区新建、改扩建中小学10所，新增学位1.2万个；教育教学质量全面提升，高考一本上线率达15.88%，高于全省3.63个百分点，我市一中学生取得了全省高考文、理科第一名的优异成绩。实施24个中央投资卫生项目和258个基层卫生项目，城乡医疗卫生服务体系更加完善。繁荣发展文化事业，公共文化设施基本实现全覆盖，文化惠民、文化遗产保护利用力度加大，全民健身、“唱响白河”等群众性文体活动蓬勃开展。“全面两孩”政策平稳实施。妇女儿童、残疾人、慈善等事业健康发展。

5.社会大局和谐稳定。防恐维稳和涉疆服务创造“南阳经验”。食品药品安全和安全生产形势总体平稳。大力推进信访法治化建设，积极化解信访积案，信访秩序明显好转。深化社会治安综合治理，努力建设平安南阳，群众安全感、满意度不断提高。

一年来，我们注重加强政府自身建设，深入开展“两学一做”学习教育，理清思路，讲究方法，注重实效，持续整治懒政怠政为官不为，强化行政监察和审计监督，加大廉政建设和反腐败工作力度，专项治理发生在群众身边的不正之风和腐败问题，干事创业氛围更加浓厚。

我们自觉接受人大法律监督和工作监督、政协民主监督、社会监督、舆论监督，认真办理人大代表建议和政协提案，支持民主党派、工商联和无党派人士参政议政。加强国防教育、国防后备力量及人民防空建设，驻宛人民解放军、武警部队和民兵预备役人员为地方发展作出新贡献。军民融合深度发展，蝉联全国双拥模范城。外事、侨务、对台、民族、宗教、统计、气象、地震、援疆等工作取得新成绩。

各位代表！成绩来之不易，前景催人奋进。这是省委、省政府和市委正确领导的结果，是市人大、市政协大力支持的结果，是全市上下齐心协力、攻坚克难的结果。在此，我代表市人民政府，向广大工人、农民、知识分子，向驻宛人民解放军指战员、武警官兵、公安民警，向所有关心支持南阳发展的各界朋友，表示崇高的敬意和衷心的感谢！

在肯定成绩的同时，我们也清醒地认识到，发展中还存在不少困难和问题：一是结构性矛盾突出。产业结构不合理，城乡区域发展不平衡，发展质量效益、财政收入结构不优。二是发展动能不足。创新能力不强，高端人才紧缺，政策环境不优，民间投资乏力，动能转换滞后。三是民生短板较多。脱贫攻坚任务艰巨，公共服务水平不高，城市功能不完善，管理水平不高，环境污染、大班额、交通拥堵等问题还很突出。四是风险隐患较多。经济下行压力较大，实体经济困难较多，经营风险、信用违约风险比较突出，防恐维稳、水质保护、安全生产等领域潜在风险不容忽视。五是政府自身建设仍需加强。政府职能转变还不到位，依法履职水平不高，一些地方、部门、基层组织和政府工作人员素质能力不适应新要求，一些领域的不正之风和腐败问题不同程度存在。六是城乡居民的道德素养、文明素养、科学文化素养仍需继续提升。对此，我们不回避、不遮掩，一定要勇于担当、认真解决。

二、2017年工作安排

今年，是党的十九大召开之年，也是落实省、市党代会精神和“十三五”规划的关键之年，扎实做好政府工作，责任重大，使命光荣。

要全面认识、准确把握基本市情和基本形势。基本市情是：人口多、面积大、底子薄、基础弱、总量大、人均少。基本形势是：发展很快、任务很重、潜力很大。发展很快：近年来，我市发展保持良好态势，进入全省第一方阵，站在了新的历史起点。任务很重：内外部环境复杂严峻，实现稳定增长困难较大；承担着保水质保生态、保发展保稳定的繁重任务，面临着转赶并重的双重压力。潜力很大：我市历史文化底蕴厚重，自然资源、人文资源、人力资源丰富，生态优良，区位优越；正处于工业化、城镇化加速期，发展韧性好、潜力足、回旋余地大；拥有卧龙综保区、京宛对口协作、中关村科技产业园、商会协会和一大批国家、省示范创建品牌等载体和平台，郑万高铁等一批重大项目相继实施，发展支撑能力显著增强；中原城市群发展规划支持我市建设区域性中心城市和区域性综合交通枢纽。尤其是，市六次党代会描绘了美好蓝图，全市上下心齐气顺、人心思进，蓄势崛起态势强劲，转型跨越前景光明。千帆竞发、百舸争流，逆水行舟、不进则退。我们要增强机遇意识、危机意识、人均意识、开放意识、竞争意识，坚定信心、振作精神，清醒忧患、加压增责，扬长避短、取长补短，在区域竞争中争取主动，在合作发展中抢占先机。

今年政府工作的总体要求是：全面贯彻党的十八大和十八届三中、四中、五中、六中全会精神，更加紧密地团结在以习近平同志为核心的党中央周围，深入贯彻习近平总书记系列重要讲话和调研指导河南工作时的重要讲话精神，深入贯彻党中央治国理政新理念新思想新战略，统筹推进“五位一体”总体布局和协调推进“四个全面”战略布局，认真贯彻中央、省各项决策部署，全面落实市六次党代会、市委经济工作会精神和“十三五”规划，适应把握引领经济发展新常态，坚持稳中求进工作总基调，坚持“四一二”工作大局和“优高稳”工作方针，突出以新发展理念为引领、以提高发展质量和效益为中心、以推进供给侧结构性改革为主线，着力推进“三去一降一补”，着力发挥优势打好“四张牌”，着力深化改革开放、推动创新，着力做到稳增长、促改革、调结构、惠民生、防风险的平衡，着力打好农村脱贫、国企改革、环境治理、发展转型、城市“双创”“五大攻坚战”，促进经济平稳健康发展和社会和谐稳定，奋力建设大美南阳、活力南阳、幸福南阳，为如期全面建成小康社会奠定坚实基础，以优异成绩迎接党的十九大胜利召开。

今年经济社会发展主要预期目标是：生产总值增长8.5%左右，规模以上工业增加值增长9%，全社会固定资产投资增长15%，一般公共预算收入增长8%左右，社会消费品零售总额增长11%，城乡居民人均收入增长8.5%以上，居民消费价格涨幅3%以下，节能减排、环境保护等约束性指标完成省下达任务。做到“四个统一”，即稳增长与调结构相统一、扩需求与促转型相统一、抓改革与防风险相统一、谋发展与惠民生相统一，使统计数字与客观形象、主观印象相一致。实现“三个同步”，即城乡居民收入与地区生产总值同步增长，生态环境质量与经济质量效益同步改善，社会事业进步与经济发展水平同步提高；实现“三个高于”，即生产总值、财政收入、居民收入增速均高于全省平均水平，加快缩小差距，使决胜全面小康、南阳转型跨越的步伐更加稳健、更加坚实。

今年政府工作的基本思路是：坚持一个优先：即民生优先。牢固树立以人民为中心的发展思想，牢记党的宗旨，坚持党的思想路线和群众路线，坚持以人为本，坚持一切为了人民，人民利益高于一切，把人民的需求作为工作重点，把人民满意作为工作标准，让人民群众拥有更多的获得感和幸福感。强化两个支撑：即强化项目建设和科技创新两个支撑。把项目建设作为工作的重中之重，以科技创新引领转型发展，强化支撑、蓄强后劲，转赶并重，实现持续快速健康发展。加快两化进程：即加快工业化和城镇化进程。以建设先进制造业强市和区域性中心城市为抓手，用好平台载体，以工业化促进城镇化、带动经济社会转型跨越。坚持四量统筹：即盘活存量、优化增量、扩充总量、提升质量。

重点抓好八项工作：

(一)强力推进产业升级，加快构建现代产业体系

打响发展转型攻坚战，深入推进供给侧结构性改革，着力“三去一降一补”，大力振兴实体经济，推动产业结构战略性调整，提升供给质量和效率。

1.加快发展先进制造业。继续实施“千百亿”产业集群和“一县一业”特色产业培育工程。以高端制造、智能制造为引领，深入推进“中国制造2025”南阳行动，制定实施年度行动计划，培育产业竞争优势。一是优化产业结构。坚定不移调高技术水平、调强制造能力、调优产业结构、调长产业链条，培育壮大装备制造、纺织服装、绿色食品三个千亿级主导产业集群，改造提升油碱化工、冶金建材等传统产业，加快发展生物医药、电子信息、新能源、新材料等新兴产业，谋划推动新能源汽车、节能环保、新型智能终端等前沿产业。研究规划汽车及零部件产业园。实施投资3000万元以上重点工业项目197个，年度完成投资235亿元。对重点产业，组建专班，出台扶持政策，建立发展基金，推动快速突破。二是培育龙头企业。实施重点企业培育计划，支持100家骨干企业做大做强，培育一批高端制造、智能制造示范企业，力争新增超10亿元企业5家、省百强企业2家、省百高企业3家。实施“专精特新”中小企业培育服务工程，培育工业“第二梯队”。三是狠抓降本增效。开展政策落实进企业活动，在减税、降费、降低要素成本上加大工作力度。盘活企业存量资产，优化负债结构，降低负债水平。以金融服务为核心做好企业服务，创新政银企沟通协调机制，开展“四项对接”，充实做大担保联盟和转贷资金池，加快推进土地证、房产证办理。抓住证监会对资本市场“直通车”机遇，推动企业直接融资，争取3家企业成功上市，30家以上企业实现挂牌。

2.加快发展现代服务业。制定实施《建设区域性商都发展规划(2017-2020)》和《促进商贸流通业发展实施意见》，实施“三个一百”行动计划，推动商贸流通、餐饮住宿等传统服务业提质发展，加快发展现代物流、电子商务、现代金融、生态文化旅游、生命健康五大主导产业，建设豫鄂陕区域性商都。一是发展现代物流业。推进国家二级物流园区布局城市建设，引进知名物流企业，大力发展冷链物流和快递业，降低物流业成本，加快推进内乡煤炭物流园、东森医药物流园、溧河物流园、铁路物流基地、西峡农商物流港等重大物流项目，加快建设区域性现代物流中心。二是发展电子商务。做好中国(郑州)跨境电子商务综合试验区南阳拓展区试点工作，实施电子商务示范创建工程，在中心城区规划建设电子商务园区。加强与知名电商战略合作，广泛开展电商培训，积极培育本土特色电商，完善电商网络体系，电子商务交易额增长50%以上，加快建设区域性电商基地。三是发展现代金融业。完善金融体系，大力引进银行、证券、基金、保险等各类金融机构，培育本土金融机构，推动金融产品和服务模式创新，加快金融街区建设，下决心培养引进用好留住金融人才，优化金融生态环境，加快建设区域性金融中心。四是发展生态文化旅游业。编制实施全域旅游规划，以“旅游+”为龙头，整合资源要素，创新体制机制，大力实施标准化建设，打造精品旅游线路，创建一批全域旅游示范县和4A、5A级景区，积极发展乡村旅游，培育旅游人才队伍，提高旅游服务质量，创新宣传推介渠道和方式，打响“‘宛’若仙境，颐养天堂”品牌，将南阳建设成为国内外知名的历史文化旅游名城。推动玉产业、玉文化转型升级、融合发展，培育新业态、新模式，打造千亿级产业集群。五是发展生命健康产业。发挥医圣故里和中医药特色品牌优势，推动中医药振兴发展，推进国家中医药综合改革试验区建设，推动医养结合，培育产业，培养人才，规划建设健康产业园区和养生养老基地，打造全国健康养生之都。

3.加快发展现代农业。深入推进农业供给侧结构性改革，大力发展农副产品精深加工，推动农村一二三产业融合发展，加快培育农业农村发展新动能，促进农业提质增效、农民持续增收。一是调整农业结构。坚持稳粮优经扩饲、农林牧渔结合、种养加一体，调优种养结构、调强加工能力、调大经营规模、调长产业链条。落实藏粮于地、藏粮于技战略，完成71万亩高标准粮田建设任务。扩大优质花生、蔬菜种植面积。坚持因地制宜、发挥优势，做大做强特色产业，突出发展以香菇、草腐菌等为重点的食用菌，以艾草、山茱萸、裕丹参等八大宛药为重点的道地中药材，以月季、玉兰等为重点的苗木花卉，以猕猴桃、薄壳核桃、软籽石榴等为重点的优质林果。以优质生猪、肉牛、奶牛为重点，依托牧原、科尔沁等龙头企业，加快发展规模高效养殖业。积极扩大香菇、月季等优势农产品出口。二是大力发展都市生态农业和有机农业。打造“一小时都市生态农业圈”，构建“三圈四带十区”都市生态农业发展格局，重点培育100个都市生态农业园区。推动都市生态农业与旅游业融合发展，加快培育休闲农业、观光农业等新型业态。坚持质量兴农，突出优质、安全、绿色导向，实施农业标准化战略，启动创建国家农产品质量安全示范市。大力发展有机农业，新发展绿色、有机农产品基地15万亩，认证绿色、有机产品30个。推进优质农产品进京，打响“水源地”和“中线渠首”品牌。三是构建新型农业经营体系。加强引导、规范运作，促进土地流转，大力发展家庭农场、专业合作社、行业协会等新型经营主体，推进区域化布局、规模化经营、标准化生产、产业化发展。引进培育龙头企业，提升农业产业化集群发展水平。新增流转、托管土地85万亩，新发展专业合作社、家庭农场600家。探索“资源变资产、资金变股金、农民变股东”模式，增加农民财产性收入。开展“互联网+”现代农业行动，建设智慧农业，大力发展农村电商，增加农民经营性收入。大力培育新型农业服务主体，创新完善农产品流通、生产服务、农业科技、金融保险等社会化服务体系，降低农业经营成本。

4.加快提升产业素质。抓好质量、管理和创新三大环节，深入推进质量强市建设，实施增品种、提品质、创品牌专项行动；引导企业对标国内外一流企业，加强全流程管理；引导企业提升创新能力，加快中高端产品开发。推进工业化、信息化融合发展，培育一批智能工厂和智能车间。加强企业家队伍建设，培养教育、关爱保护、帮助支持企业家，动真情、解难题、办实事，做到亲商、安商、扶商，营造“亲”、“清”政商关系，造就一支敢为人先、精益求精、诚信尽责的企业家队伍，以企业家素质提升引领产业素质提升。

(二)强力推进城乡一体化，加快新型城镇化进程

坚持遵循规律、以人为本、规划引领、有序推进，构建中心城区、县城、重点镇、美丽乡村四级城镇体系，探索具有南阳特色的生态、融合、适度、集约、高效城镇化路子。

1.下功夫提升中心城区首位度。发挥中心城区在城镇化中的龙头作用，突出“水、绿、文、产”特色，更加注重城市内涵建设和管理水平提升，建设宜居宜业区域性中心城市。一是强化规划引领。依法制定城市规划。加强规划编制和审批管理，坚持创新理念、改进方法、公众参与、严格程序，把以人为本、尊重自然、传承历史、绿色低碳等理念融入城市规划全过程，增强规划的前瞻性、严肃性和连续性，实现一张蓝图干到底。坚持远近结合、适度超前，做好“十三五”城市近期建设规划、高铁片区控制性详细规划、城市设计、核心区修建性详细规划等重点规划编制工作，做好城市重点地段城市设计。坚持协调发展理念，加强空间开发管理，引导调控城市规模，优化城市空间布局和形态功能，确定建设约束性指标。对各类建设项目，必须严格按国家法律法规和省、市政策要求，同步规划建设公共设施，非商业区不得新建、改建、扩建商业“门面房”。严格依法执行规划。强化规划的龙头带动、战略引领、刚性控制和强制性。加快编制控制性详细规划，未编制控制性详细规划的区域，不得进行建设。完善规划委员会制度，健全监督、考核、社会参与和公开、报告制度，规划面向社会公开，吸收公众参与，接受社会监督，改进监控手段，实现全天候、全方位、全覆盖、常态化监管。任何单位和个人都必须严格执行规划，严格依法用地和建设。党员干部要作守法护法、抵制违法的模范。加强普法宣传，使城市规划建设管理的法律法规家喻户晓、人人皆知、深入人心，让违法行为无藏身之地。坚决遏制新增违法建设，全面清查处理违法用地、违法建设行为，对违法建设行为要严格依法处理，该拆除的拆除、该没收的没收，坚决杜绝以罚代管，重点查处“特殊人”、“钉子户”。同时，要严厉查处违法用地、违法建设背后的失职渎职和官商勾结、以权谋私行为。通过查处违法用地、违法建设行为，回应群众关切，化解突出矛盾，净化社会风气，强化规划意识、法治观念，规范房地产市场。二是强化土地管理。严格土地用途管制制度，控制建设用地增量，盘活存量土地，依法清理闲置土地，强化节约集约利用土地。加强对新修道路、河道、学校及广场公园周边的建设控制和土地收储，牢牢控制土地一级市场，增强宏观调控能力，规范土地市场运作，使财政投入的增值收益回归财政和公众。全面实行净地加规划设计条件出让，对城市功能有重大影响或建筑景观有特殊要求的重要地块，实行净地加修建性详细规划出让。三是开展房地产市场专项整治。我市房地产领域问题较多，主要表现为房地产企业数量多、实力弱、诚信差，房地产项目手续不齐或没有手续、违法占地、违法建设，小区规划设计建设档次低、环境差。上述问题，破坏了城市规划、扰乱了市场秩序、侵害了群众利益、影响了发展大局，社会反映强烈。必须以更大的决心、更硬的措施，强力推进房地产市场专项整治。坚决禁停所有违法建设，全面排查、澄清违法底子，依法依规、分类处理、从严惩处，不留后遗症。建立房地产市场准入清出和联动制约机制，引进、培育一批优质房地产企业，引导带动我市房地产市场步入良性发展轨道。四是完善城市功能。研究论证、科学制定城市重点建设工程项目三年计划，分清轻重缓急，逐年组织实施。要换位思考，设身处地替群众着想，规范征地拆迁行为，保护群众合法权益。实施城市道路建设工程，加快推进中心城区外环路建设，开工建设南阳至官庄、鸭河、社旗、镇平快速通道，研究规划南阳到镇平、官庄、唐河、鸭河的轨道交通；以完善路网结构、打通断头路为重点，实施北京路南延、独山大道北延等22项市政道路建设项目，开展重点区域立体交通体系前期研究；积极争创公交都市，新增一批新能源公交车，建设公共自行车交通系统，规划建设一批公共停车场，构建外连内畅的城市交通体系，解决群众出行难问题。实施内河综合治理工程，全面完成温凉河综合治理工程，基本完成梅溪河、三里河、溧河综合治理工程，完成十二里河、汉城河截污和护城河水体生物修复工程，把内河打造成为水清景美的城市新亮点。实施公用设施建设工程，南水北调配套龙升水厂、麒麟水厂建成运行，新建、改造城市供气管网71公里，新建供热管网20公里、集中供热率达到45%，市污泥处置厂建成投运，加快推进污水处理厂三期工程。建成城市地下综合管廊6.5公里。实施城市绿化美化工程，抓好公园游园、河道绿化、道路绿化等工程建设，积极创建国家生态园林城市，让环境更加优美、城市更加靓丽。完成海绵城市试点年度任务。五是提升管理水平。今年是“双创”决胜之年，我们要摆出决战姿态，树立必胜信心，广泛宣传发动，严格对照标准，严控时间节点，全面查缺补漏，成功创建国家卫生城市、全国文明城市。全市动员、四级联动，积极创建国家级、省级卫生县城(村镇)、文明县城(村镇)。要坚持“双创”重在过程、重在持续、重在提升的理念，抓基层、抓基础，抓根本、抓机制，加强城市管理和服务体系智慧化建设，促进大数据、物联网、云计算等现代信息技术与城市管理服务融合，提升城市精细化管理水平，确保城市品位、文明程度持续提升。

2.全面推动城乡一体化发展。把中小城市、县城、中心镇作为新型城镇化的重点。一是完善县域城镇体系。全域推进百城建设提质工程，围绕完善县域规划体系、提高综合承载能力、推进产城融合发展、提高人居环境质量、提升城市精细化管理水平等关键环节，搞好土地储备，纳入财政预算，组建合资公司，做实申贷要件，力争用3～5年时间，建成和谐宜居、富有活力、各具特色的县域城镇体系。加快县城建设，统筹地下地上建设，谋划推进水电路气暖、垃圾污水处理等基础设施和教育、医疗卫生、文化体育等公共服务项目，推进产城融合，打造宜居环境，全面提升县城承载能力和发展水平。加快小城镇发展，重点培育60个重点镇、特色镇、宜居美丽小镇。推进新农村建设，深入开展农村人居环境治理，建设美丽宜居乡村。扎实推进新型农村社区遗留问题整改。二是推进组团发展。推动市中心城区与官庄、鸭河、唐河、社旗、方城、镇平相向发展，加快交通一体、产业配套、服务共享，培育卫星城镇，加快形成“一心六组团”南阳都市区。三是健全城乡发展一体化体制机制。持续实施“一基本两牵动三保障”，深化户籍制度改革，加快构建以居住证制度为载体的基本公共服务供给机制，维护进城落户农民土地承包权、宅基地使用权、集体收益分配权，加快落实农村宅基地复垦券制度。落实城镇建设用地增加与吸纳农业转移人口落户数量挂钩政策。深入推进市城乡一体化示范区管理体制改革创新，积极推进西峡、社旗、唐河新型城镇化试点工作，实施千村城乡一体化示范工程。

(三)强化项目支撑，加快培育新的经济增长点

项目关乎发展，项目决定后劲。要把经济社会发展各项任务落实到项目上，把全市上下的力量凝聚到项目上，全面掀起新一轮项目建设热潮，以项目建设大突破支撑南阳新发展大跨越。

1.明确目标。编制实施项目建设五年规划和年度计划，分清轻重缓急，做到投产达产扩建一批、开工建设一批、谋划论证争取一批，压茬推进、滚动实施，力争每年实现“三个三分之一”，即重点项目、实体经济项目、工业项目年度实际完成投资均增长三分之一以上。

2.把握方向。一是结构调整项目(包括农副产品、矿产品、劳动密集型等资源开发利用深加工项目；“三高四新”项目，即高科技、高附加值、高市场占有率项目，新能源、新材料、新产业、新业态项目；产业补链延链强链项目)；二是强基础、补短板、惠民生和生态、文化、旅游项目。

3.突出重点。在区域上：突出市区、县城、产业集聚区和服务业“两区”；在主体上：发挥好上级投资、现有企业、“回创”人才、招商引资“四个主体”作用；在重点项目上：抓大不放小，每年突出抓好“四个三十”，即30个重大建成项目、30个重大在建项目、30个重大开工项目、30个重大储备项目。

4.务求实效。加强对项目工作的领导，一把手亲自抓项目，单位无空档、时空无空档，上下联动、条块结合，组建专班、全员参与，强力推动。建立项目清单，明确时间节点，逐项分解任务，层层传导压力。建立重大项目金融机构参与论证机制，全面落实领导分包、联审会商等制度，每月集中会诊，定期现场办公，及时解决问题。对“四个三十”项目，实行一周一碰头、一月一研究、一季一述职点评、半年一观摩，加强考评、真实考评、严格奖惩，形成全市上下凝神聚力抓项目的氛围和导向。

(四)强化科技创新，加快培育新动能和核心竞争力

实施科技强市核心战略，以科技创新改造提升传统产业、催生新产业新业态，加快动能转换，促进转型升级。

1.培育主体。支持自主创新，强化企业主体地位，深入实施高新技术企业和“科技小巨人”企业培育工程，推进大中型企业省级以上研发机构全覆盖，引导企业瞄准高端、前沿、重点领域，攻克一批关键技术。实施开放创新，引进创新人才、技术设备、管理经验、先进理念，加强与省科技厅、省农科院、郑州大学、西北农林科技大学、北京农学院等单位的务实合作；大力推进军民融合协同创新，争创国家级军民融合创新示范区。推动大众创业万众创新，完善政策体系和支撑平台，培育壮大创客群体。认真落实“南阳人才新政40条”，实施高端人才引进、紧缺型人才培育计划，深入推进全民技能振兴和职教攻坚工程，建好领导干部、管理人员、专业技术人员、熟练技能人才“四支队伍”，营造良好的人才发展环境，实现人尽其才、才尽其用。加强科普工作，推广实用技术，提高全民科学素质和科技创新能力。

2.提升平台。集聚资源要素，把高新区、市城乡一体化示范区、产业集聚区培育成为科技创新的主阵地。突出抓好与中关村的深度合作，建好用好中关村科技产业园和京宛科技合作平台，努力把南阳建设成为中关村科技成果的孵化、转化基地。深度对接融入郑洛新国家自主创新示范区，争取实现资源共用、政策共享。支持南阳师范学院、南阳理工学院等高校创新平台建设。大力发展楼宇型、街区型、小镇型众创空间，加快南阳创业大街二期等项目建设，着力培育15个特色小镇，实现创新创业平台县区全覆盖。持续实施“百项科技创新项目”，重点实施生物质能源、功能性复合材料、生物制药、医疗器械、智能终端等五大创新发展行动。新增省、市重点实验室、工程技术研究中心20家以上。

3.完善机制。市级设立3000万元专项资金，实施流资贷款贴息，支持符合条件的高新技术企业发展。加大研发投入、力争占生产总值的比重达到全省平均水平。深化科技管理体制改革，创新分配机制，保护知识产权，加快政府职能由研发管理向创新服务转变。健全“产学研用金”协同创新机制，提高创新效率。

4.创新业态。加快发展大数据、云计算、物联网，推动数据资源开放共享和开发应用。深入实施“互联网+”行动计划，全面推进互联网与经济社会融合发展，促进产业变革和商业、服务、管理模式创新。积极培育枢纽经济、创意经济、生态经济等融合发展新业态。

(五)着力深化改革开放，增强发展新活力

破解体制障碍，强化开放带动，优化发展环境，激发活力动力。

1.持续深化改革。聚焦影响经济社会发展和民生改善的突出矛盾和问题，深化重点领域和关键环节改革，使改革精准对接发展所需、基层所盼、民生所向。一是深化国企改革。完善国资监管体制，基本完成企业改制遗留，推进驻宛央企“三供一业”分离移交工作。二是深化财税金融和投融资体制改革。认真落实预算管理体制和税制改革，推动政府投融资平台市场化转型，扎实做好财政性涉企资金基金化改革，加快推进农信社改制组建农商行。推广政府和社会资本合作(PPP)模式，切实用好专项建设基金、央企和大企业集团等领域资金。三是深化农业农村改革。全面完成土地承包经营权确权登记颁证，开展房地一体的农村权籍调查，颁发统一的不动产权证书，落实承包土地所有权、承包权、经营权分置政策。四是推进社会民生领域改革。全面完成全国社会信用体系建设示范市年度创建任务。加快推进医药卫生、收入分配、价格、文化等领域改革。

2.全面扩大开放。解放思想、抢抓机遇，实施开放带动，拓展发展新空间。一是用好开放平台。积极对接中国(河南)自由贸易区、郑州航空港经济综合实验区等重大开放平台，强力推动卧龙综保区完善功能、集聚企业、培育产业。谋划打造铁路、航空一类口岸，以大通关促进大开放。推动外贸转型升级，实现进出口总额17亿美元。积极参加国家、省组织的重大招商活动。以筹办世界月季洲际大会为动力，加快推进“一主两副”月季园和30个月季游园规划建设，办好2017年南阳月季展，编制月季产业发展规划，提升发展水平，打造世界月季名城。二是寻求开放合作。依托各地商会，抓住京宛对口协作这个大平台，对接京津冀，在高科技产业、科教卫生、文化旅游、人力资源等方面全方位加强合作，力争引进一批知名企业、学校、医院和科研院所。积极参与区域合作，主动融入长江经济带，研究出台融入“一带一路”行动计划，加快推进南阳欧洲工业园建设。加强与国内外知名企业战略合作，积极发展总部经济。支持优势企业走出去。三是推动开放招商。制定务实管用的招商引资政策措施，突出重点区域，积极组织产业链招商、定向招商，运用好股权、并购等招商新模式。抓好招商项目落地，提高项目履约率、资金到位率、项目开工率。更加注重招优引强，高污染、高耗能、低附加值的项目坚决不上。

3.优化发展环境。环境是生产力，决定竞争力。深化“放管服”改革，完善权力清单，精减行政审批事项，清理规范中介服务；深化商事制度改革，探索试行“多证合一”。完善责任清单，强化事中事后监管，全面推行“双随机、一公开”监管模式，探索建立市场监管、综合执法新机制。完善效率清单，优化便民服务网络，推广“互联网+”政务服务、投资项目审批线上办理、联审联批、全程代办等审批服务模式，开展减证便民专项行动，设身处地、换位思考，精准对接、优质服务，让群众和企业少跑腿、好办事、不添堵。着力营造市场化、国际化、法治化的营商环境，坚持一视同仁，实行国民待遇，重合同、守信用、抓落实。完善鼓励投资的政策体系，规范政府投资行为和公共资源交易行为，依法从重从快打击阻工闹事、强揽工程、强买强卖等行为，保护投资者合法权益。

(六)着力强化基础能力，增强发展新动能

坚持突出重点、整体推进，弥补短板、提升功能，强化基础支撑，提升综合竞争优势。

1.推进重大基础设施建设。围绕建设区域性综合交通枢纽，加快推动重大交通基础设施建设。科学完善加密航线航班，开展南阳机场迁建前期工作，开工建设淅川通用机场，加快鸭河、镇平等通用机场前期工作；强力推进郑万高铁、蒙华铁路建设，积极开展宁西高铁南阳—信阳—固始段和呼南高铁豫西通道前期研究工作，南阳火车站改扩建工程2018年春运前基本建成；加快周南、栾西等高速公路和环丹江口库区旅游公路建设，开工建设西十高速、方枣高速、唐河复航等项目；加强干线公路和县乡公路建设，建设农村公路2500公里。加强信息基础设施建设，推进“全光网南阳”全面升级，完成“宽带中国”示范城市建设任务。坚持政府主导、统筹规划、整合资源，加快建设智慧城市。构建绿色高效能源保障体系，加快内乡煤电运一体化、南召天池抽水蓄能电站等重大能源项目建设；大力发展光伏发电和风电；继续实施新一轮农网改造升级工程。加快推进内河水源工程二期等水利工程建设，启动罗汉山大型水库前期工作。

2.完善提升科学发展载体。推动产业集聚区提质转型发展，推进有条件的产业集聚区晋升为国家级、省级经开区、高新区，提升产业集群发展水平。全市产业集聚区力争完成固定资产投资1700亿元，实现规模以上工业主营业务收入2700亿元。推动服务业“两区”提速增效发展，加快产业集聚、特色培育和功能提升。探索“政府引导+市场化运作”模式，处置盘活闲置资产，推进产业集聚区和服务业“两区”二次创业、创新发展。

(七)着力改善生态环境，增强发展新优势

牢固树立“绿水青山就是金山银山”的强烈意识，推进国家生态文明先行示范区建设，倡导绿色低碳循环发展理念，加强环境保护，厚植生态优势，实现更高质量、更有效率、更加公平、更可持续的发展。

1.持续加强环境治理。坚持依法治理、标本兼治、疏堵结合，推动环境质量持续改善，让人民群众呼吸上新鲜空气、喝上干净水、吃上放心食品，在优美环境中享受美好生活。一是继续开展大气治理攻坚。实施预算式管理，推进科学治污、依法治污、全民治污，落实“六控一应对”，确保PM10浓度降到每立方米102微克以下，PM2.5浓度降到每立方米65微克以下，空气优良天数达到205天以上。二是打响水污染治理攻坚战。推进水生态文明城市建设，以确保水质达标、治理黑臭水体、保障水源安全为重点，实施“五水共治”，全面落实“河长制”，一河一策、精准治污、水陆统筹，系统推进水污染防治、水生态保护和水资源管理，加快全市内河治理步伐，确保水环境质量持续改善，确保丹江口水库水质和城市集中饮用水水源稳定达标。三是积极推动土壤污染防治。启动土壤污染状况详查，加强土壤污染治理与修复，推进农业面源和重金属污染防治，严格危险废物环境监管。严厉打击环境违法犯罪行为，坚决让污染环境、破坏生态者付出沉重代价。

2.推进生态工程建设。坚持“山水林田湖是一个生命共同体”理念，推进森林、湿地、流域、农田、城市五大生态系统建设。以创建国家森林城市为抓手，持续开展大造林大绿化，见缝插绿，适地适树，突出乡土树种和高大乔木，做到乔灌花草相搭配，创新机制，明晰产权，提高造林质量。优化城市绿地布局，构建绿道系统，实现城市内外绿地连接贯通。重点实施南水北调中线生态廊道、白河生态防护林带、高速通道等绿化工程，持续抓好丹江口库区、鸭河口水库、淮河源头等重要水源区生态林建设，加快国家储备林基地、白河国家湿地公园、紫山植物园等项目建设，完成营造林183万亩。

3.完善生态文明制度体系。科学划定生态保护红线。积极推进地方生态环境保护立法，研究出台实施细则。建立完善控制污染物排放许可制度。开展用能权有偿使用和交易试点。强化能源消费、水资源消耗总量和强度控制，实施最严格的水资源管理制度。大力发展循环经济，启动建设固废园区，加快静脉产业示范园区建设。坚持统筹规划、整体招商、全域推进，力争用三年时间实现全市生活垃圾无害化处理、资源化利用。

(八)着力维护和谐稳定，增强人民新福祉

牢固树立以人民为中心的发展思想，优化基本公共服务供给，集中力量办好重点民生实事，致力解决就学、就医、就业、社保、安全等现实问题，进一步提升群众的获得感幸福感。

1.打好脱贫攻坚战。把脱贫攻坚放到经济社会发展全局重中之重的位置，坚持精准谋划、精准识别、精准施策、精准退出、精准考核，突出抓好产业扶贫、易地搬迁、社会兜底、教育培训和基础设施建设，推进农村低保制度与扶贫政策有效衔接，提高贫困地区和贫困群众自我发展能力，统筹做好非贫困县贫困村和非贫困村贫困户的脱贫工作，确保12.3万贫困人口稳定脱贫，方城县实现脱贫摘帽。把脱贫攻坚与致富奔小康结合起来、与城乡一体化建设结合起来。

2.做好就业和社会保障工作。突出做好农村富余劳动力、高校毕业生、去产能企业分流职工三大群体就业工作，积极做好军改期间军队转业干部、随军家属、退役士兵接收安置工作。促进“零就业家庭”成员实现就业，完善落实就业创业各项扶持政策，支持大学生和返乡农民工创业。新增各类就业13.8万人。扩大社会保障覆盖面，实施全民参保登记计划，稳步提高社会保障待遇水平，深化机关事业单位养老保险制度改革，实施统一的城乡居民基本医保制度，落实困难群众大病补充医疗保险制度。完成棚户区改造2480套、农村危房改造4500户。加快发展养老、社会福利和慈善事业，市级养老中心和10个县级福利中心建成投用。关注南水北调移民后续发展致富。完善社会救助体系，建立扶贫济困机制，落实社会救助标准与物价上涨挂钩联动机制和特困人员供养制度，加强农村“三留守”人员关爱服务和困境儿童保障，让特殊群体感受到社会的关爱和温暖。

3.努力办好教育事业。百年大计，教育为本。坚持端正理念、科学管理、软硬结合，做到教书育人、以文化人、身心健康，高标准、高起点、高水平、高效率推动教育事业发展。统筹谋划，整合资源，持续实施“全面改薄”和扩充城镇义务教育资源五年计划，致力改善农村办学条件，重点抓好农村中小学校优化布局和农村寄宿制学校建设，实施乡村教师激励政策，对县区实行目标管理，强力推进义务教育均衡发展。开工建设南阳一中新校区，推动第一批4所15年制完全学校建设，新建、改扩建中小学6所，新增学位2万个以上；加快推进职教园区建设，强力整合现有中专、干校、技校等闲置职教资源，扩大中小学校办学规模，致力解决中心城区入学难、大班额问题。统筹发展学前教育、高中教育、高等教育和特殊教育。积极引进名校开展合作办学，支持规范民办教育发展。深化教育领域综合改革，提高管理人员和教职工素质，提升教育教学质量。开展希望工程、春蕾计划等爱心助学活动，决不让贫困把孩子阻挡在学校大门之外。

4.推动健康南阳建设。没有全民健康，就没有全面小康。实施医疗高地、中医药强市、医养结合“三大工程”，坚持预防为主、防治结合、重心下移，政府与市场共同发力，加快中心医院示范区分院、市骨科医院、市口腔医院等重点项目建设，推进基本卫生服务均等化，加强农村医疗卫生体系建设，逐步实现分级诊疗，努力解决看病难、看病贵问题。推动人口和计划生育工作转型发展。广泛开展全民健身活动。

5.繁荣发展文化事业。加快构建现代公共文化服务体系，加强基层综合文化服务中心建设。深入开展“唱响白河”等群众文化活动。加大文物保护力度。创新发展文化产业，培育一批文化产业园区、骨干企业和品牌。

6.做好安全稳定工作。树立“万无一失、一失万无”理念，认真落实“防范、团结、服务、管理、处置”工作方针，牢牢把握防恐维稳主动权。严格防控金融风险，严厉打击和处置非法集资，妥善化解实体经济经营风险，切实加强企业互保等领域风险防控。建立排危除险机制，全面排查治理各类安全隐患，严格落实安全生产责任制。加强防灾减灾救灾体系建设，严管食品药品安全，强化社会治安综合治理，维护群众衣食住行和生命财产安全。依法处置信访问题，全面治理拖欠农民工工资问题，加强舆情引导管控，确保社会大局和谐稳定。

加强精神文明建设。千百年来，南阳大地上孕育出了以“四圣”、召父杜母、范仲淹、彭雪枫、冯友兰等为代表的古今仁人志士和好人群体，他们身上所体现的躬耕奉献精神、家国情怀和民本、忧乐、清廉思想，是留给南阳的一笔宝贵财富，更是激发我们振兴南阳的强大动力。我们要把传承优秀传统文化与弘扬社会主义先进文化结合起来，与弘扬社会主义核心价值观和移民精神、农运会精神、雷锋精神结合起来，广泛开展学习英雄王锋、志愿者服务、送温暖献爱心、南阳好人评选表彰等群众性精神文明创建活动，讲好南阳故事，推动移风易俗，倡树文明新风，全面提升城乡社会文明程度和群众文明素质，在全市上下形成崇德向善、人心思进的浓厚氛围。

加强国防教育和国防后备力量建设，支持驻宛人民解放军、武警部队、民兵预备役和人民防空建设，持续开展双拥工作，推进军民融合发展，巩固军政军民团结。广泛开展法治宣传教育，做好法律援助和社区矫正工作。支持工会、共青团、妇联、工商联等人民团体更好发挥桥梁纽带作用。做好外事、侨务、民族、宗教、对台、统计、史志、档案、地震、援疆等工作。

三、加强政府自身建设

按照忠诚干净担当、为民务实清廉的要求，坚持以民为本、以实为魂、以干为乐、以廉为荣，加强学习、加强实践、加强修养，努力建设勤政廉洁法治高效政府。要对人民负责，经得起历史检验。

(一)勤政为民。牢记宗旨，时刻把人民的冷暖疾苦放在心上，把人民的意愿和需求作为出发点，把人民的满意作为评价标准，让人民赋予的权力始终用来为人民服务。奋发有为，保持拼搏竞进、争先创优的精神状态，担当作为、勤勉敬业，尽职尽责、尽心尽力。务实重干，想干、会干、实干，多做打基础、管长远、利民生的工作，决不搞形象工程、政绩工程，严控“三公经费”，把有限的财力用到推动发展、改善民生上。

(二)清正廉洁。严守政治纪律和政治规矩，深化“两学一做”学习教育，增强“四个意识”特别是核心意识、看齐意识，自觉遵守《准则》、《条例》，严格落实中央八项规定精神和省、市若干意见。坚持从严治政，落实主体责任和“一岗双责”，大力推进政府系统党风廉政建设，坚决整治侵害群众利益的不正之风和腐败问题，以反腐倡廉实效保障发展、取信于民。自觉接受监督，加强党内监督，发挥行政监察、审计监督和社会监督作用，全面推进政务公开，让权力运行更加公平公正、阳光透明。

(三)依法行政。全面落实法治政府建设实施纲要，落实依法行政一把手责任制，把政府工作全面纳入法治轨道。推进依法科学民主决策，落实法律顾问制度，加强新型智库建设，坚持问题导向，善于辩证思维和不可行性研究，敢于纠偏纠错，落实重大决策终身责任追究及责任倒查机制。坚持公正文明执法，严格加强执法监督。自觉接受市人大及其常委会的法律监督、工作监督，主动接受市政协的民主监督，坚决执行人大及其常委会的决议决定，认真办理人大代表建议和政协提案，广泛听取民主党派、工商联、无党派人士、人民团体和社会各界人士意见。

(四)提升效能。提升能力素质，强化学思践悟。要善于分析形势、把握大势，研究政策、运用政策，用心动脑、想清悟透，形成读书学习的风气，定期开展法治、金融、产业等业务培训，努力成为专家型干部和行家里手。讲究工作方法，运用好把脉问诊、对症下药，统筹兼顾、突出重点，典型引路、点面结合，因地制宜、分类指导，专班推进、分工协作，分级负责、层次领导，宣传引导、发动群众等基本工作方法。转变作风，贵在坚持、重在落实，要以踏石留印、抓铁有痕的实劲狠劲，发扬钉钉子精神，久久为功、善作善成，做到出工、出力、出效、出彩。务求实效，尽可能减少不必要的会议、文件、活动，集中力量抓项目、抓落实。

各位代表！决胜小康、圆梦中华、中原出彩、南阳振兴，匹夫有责、时不我待。让我们更加紧密地团结在以习近平同志为核心的党中央周围，在省委、省政府和市委的坚强领导下，紧紧团结、依靠和带领全市人民，牢记使命、不负重托，撸起袖子加油干，加快转型跨越发展，为建设大美南阳、活力南阳、幸福南阳，为全面建成小康社会打下坚实基础，以优异成绩迎接党的十九大胜利召开！
